# Acute limb ischemia in a patient with cardiac amyloidosis: a case report

**DOI:** 10.4076/1757-1626-2-8525

**Published:** 2009-09-08

**Authors:** Christos Verikokos, Marika Moschou, Evridiki Karanikola, Stephanie Vgenopoulou, John Bellos, Panagiotis Roukanas, Efthimios Avgerinos

**Affiliations:** 1 Second Department of Propedeutic SurgeryAgiou Thoma 17, 115 27 Goudi, “Laiko” General Hospital, Medical School, University of AthensGreece; 2 A’ Laboratory of Pathologic AnatomyAgiou Thoma 17, 115 27 Goudi, “Laiko” General Hospital, Medical School, University of AthensGreece

## Abstract

**Introduction:**

Cardiac amyloidosis is a manifestation of several systemic diseases known as amyloidoses. Arterial thromboembolic complications have not been reported to occur frequently, although the pathophysiology of cardiovascular amyloidosis would theoretically predispose to such manifestations.

**Case presentation:**

We present the case of a 52-year-old woman, who suffered from cardiac amyloidosis and was admitted to our hospital for left acute limb ischemia. An urgent embolectomy was performed, improving her clinical condition and the pathologoanatomic examination of the embolus revealed deposition of amyloid.

**Conclusion:**

Peripheral arterial thromboembolic events in patients with amyloidosis are rare. An antiplatelet treatment is recommended in such patients with cardiac amyloidosis for the prevention of embolism.

## Introduction

Amyloidosis, despite being a single entity, is a general term covering a wide range of variable diseases, quite though uncommon. Data from Olmsted County, Minn, reflect age-adjusted incidences between 6.1 and 10.5 per million person-years [1]. It is estimated that 1275 to 3200 new cases occur annually in the United States [1-3]. Amyloid disposition can be found in any part of the body associated with variable non-specific symptoms. Particularly though cardiac involvement is one of the causes which can lead to death. The usual pathophysiology involves myocardial infiltration producing slowed diastolic filling. Less frequently, cardiac amyloid may simulate cardiomyopathy, congestive heart failure, coronary heart disease, valvular heart disease or arrhythmia. Arterial thromboembolism is an unusual phenomenon in cardiac amyloidosis. Investigation of contributing causes reveals disorders producing stasis, endothelial disturbance and probably abnormalities in blood coagulability [4]. We herein present a patient with amyloid heart disease complicated by cardiogenic systemic arterial thromboembolism.

## Case presentation

**Figure 1.**
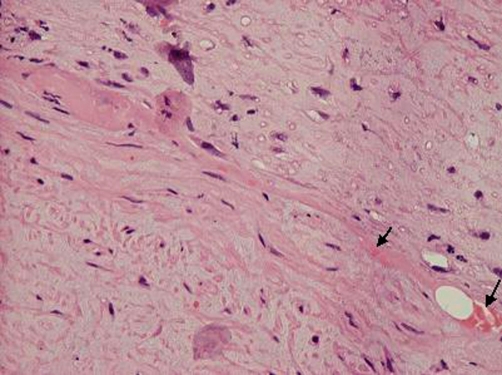
Partial deposition of amorphous eosinifilic substance in the vascular wall (H&E ×400).

**Figure 2.**
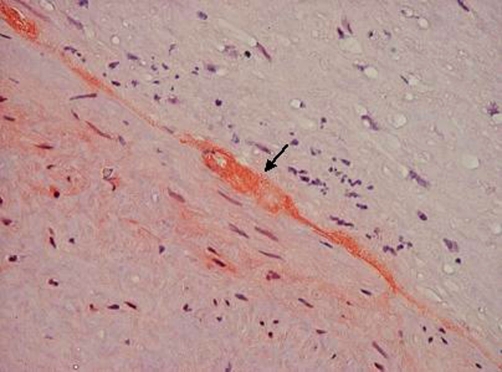
Deposition of amorphous eosinifilic substance, double direction in polarized light (coloured: Congo Red × 400, compatible with amyloid).

A 52-year-old Greek woman was transferred to the Emergency Unit of our Hospital with symptoms of acute left-limb ischemia. The patient reported sudden onset of calf pain, starting five days before. Symptoms rapidly deteriorated on admission day. The clinical examination revealed a cold, pale and painful left leg with absent peripheral pulses but normal ones on the right. The patient was in good general condition, the pulse was 67/min, the arterial blood pressure was 100/50 mmHg and lung examination did not reveal any particular findings. The EKG showed low voltage without atrial fibrillation. Her past medical history indicated cardiac insufficiency due to amyloidosis. A past echocardiogram had revealed restrictive cardiomyopathy with hypertrophy of the left ventricular wall and two years ago myocardial biopsy had shown positive histochemical stains for red of Congo and positive immunohistochemical staining for serum amyloid P component and lamda light chains indicative of primary amyloidosis (AL). As the biopsy was performed elsewhere, adequate information for the site of ventricle punctured, do not exist. An urgent embolectomy was performed following intravenous administration of 5000 IU heparin. Histology of the embolus revealed amyloidosis (Figure 1 & Figure 2). Postoperatively, intravenous heparin and per os anticoagulants (warfarin) were administered. Alongside, low doses of inotropes were required to maintain adequate blood pressure. A new echocardiogram revealed good myocardial function with diastolic dysfunction, calcification of the valves, mild mitral, tricuspidal and aortic valve insufficiency, while also hypertrophy of the left ventricle and restrictive amyloid cardiomyopathy. The patient was discharged on the 6th postoperative day in good general condition. Three months later oral anticoagulants were discontinued and low dose of aspirin (100 mg/day) was prescribed. To date the patient has not presented any type of recurrence.

## Discussion

The exact mechanism by which aggregation of amyloidosis causes damage and consequent dysfunction has been widely studied and discussed. The cardiovascular system is among the common targets of amyloidosis [5]. In clinical practice, amyloidosis is categorized in primary, secondary and hereditary. Primary (idiopathic, systemic) presents without previous or coexisting disease; it may involve the cardiovascular system, the gastrointestinal tract and the muscles. Secondary amyloidosis is linked to chronic diseases and has a tendency to target parenchymal organs such as the liver, spleen, and kidneys [6]. Regarding the heart symptoms of amyloidosis are not concrete. Cardiac amyloidosis can mimic cardiomyopathy, coronary cardiac disease, valvular cardiac disease or arrhythmia. The most common clinical feature though is right cardiac insufficiency, while coronary artery disease manifests predominantly in men by 60-65%, and only 1% of patients are younger than 40 years [7]. Concerning the natural history of thromboembolic disease in patients with amyloidosis, the medical files of 2.132 patients were evaluated in the Mayo Clinic between 1975 and 2000 and forty patients (21 men, median age 65) were selected to objectively evaluate the incidence of thromboembolic disease. Twelve patients had cardiac amyloidosis and 20 had kidney amyloidosis. Neither the extent of amyloidosis nor the type of monoclonal protein was predictive of thromboembolism. Thromboembolism manifested before the diagnosis of amyloidosis in 11 patients, during the diagnosis or within a month after the diagnosis in 11 patients, and one month or more following diagnosis in 18 patients. Twenty nine patients (73%) had vein thrombosis and 11 (28%) had arterial thrombosis. Eight patients (20%) died within a month after the thrombotic formation, and 18 (45%) died within a year. Thromboembolic events in patients with AL amyloidosis anticipated a significant mortality within the first year following the event [8]. The patients with cardiac amyloidosis presented a cardiac insufficiency with impaired diastolic function despite satisfactory systolic function. Such patients, though rarely, can develop cardiogenic thromboembolic disease. The rarity of this complication is impressive taking into consideration the pathophysiological basis of cardiac amyloidosis. Research into the contributing causes reveals that events present on the basis of the classic Virchow triad involving disturbance of blood flow, endothelial damage and possible abnormalities of blood coagulability. These facts lead to concrete proposals for prophylaxis and management of the diseased population [9].

Cardiac amyloidosis is considered a potential cause of systemic embolism. Nevertheless, such events have not been frequently reported. Cardiac mural clot is common in autopsies of patients with amyloidosis. Recommended diagnostic assessment is transoesophageal cardiac ultrasound to investigate clots within the endocardium. On such high likelihood of thromboembolic episodes, anticoagulation treatment as a preventive measure should be considered. Low-dose aspirin appears to be safer for thromboprofylaxis than the use of warfarin [4].

## Conclusions

Even if amyloidosis is a complex disease, it complies with the triad of Virchow and predisposes the patients to thromboembolic events. This case report presents such an infrequent incidence in a patient who suffered from cardiac amyloidosis complicated by a thromboembolic episode of the lower limb. For such cases, we advocate thromboprofylaxis with low-dose (100 mg/day) aspirin. In patients with atrial fibrillation or arrhythmias we should consider administering anticoagulants (e.g. vitamin K antagonists) in therapeutic doses (INR 2.0-2.5).

## Abbreviations

AL: amyloid light chain
INR: international normalized ratio

## References

[bib-001] Feng DL, Edwards WD, Oh JK, Chandrasekaran K, Grogan M, Martinez MW, Syed II, Hughes DA, Lust JA, Jaffe AS, Gertz MA, Klarich KW (2007). Thrombosis and embolism in patients with cardiac amyloidosis. Circulation.

[bib-002] Kyle RA, Linos A, Beard CM, Linke RP, Gertz MA, O’Fallon WM, Kurland LT (1992). Incidence and natural history of primary systemic amyloidosis in Olmsted County, Minnesota, 1950 through 1989. Blood.

[bib-003] Falk RH, Comenzo RL, Skinner M (1997). The systemic amyloidoses. N Engl J Med.

[bib-004] Browne RS, Schneiderma H, Kaıpini N, Radjlrd MJ, Hager WD (1992). Amyloid heart disease manifested by systemic arterial thromboemboli. Chest.

[bib-005] Gertz MA, Lacy MQ, Dispenzieri A, Hayman SR (2005). Amyloidosis. Best Pract Res Clin Haematol.

[bib-006] Kholova I, Niessen HWM (2005). Amyloid in the cardiovascular system. J Clin Pathol.

[bib-007] Falk RH (2005). Diagnosis and management of the cardiac amyloidoses. Circulation.

[bib-008] Halligan CS, Lacy MQ, Rajkumar SV, Dispenzieri A, Witzig TE, Lust JA, Fonseca R, Gertz MA, Kyle RA, Pruthi RK (2006). Natural history of thromboembolism in AL amyloidosis. Amyloid.

[bib-009] Hausfater P, Costedoat-Chalumeau N, Amoura Z, Cacoub P, Papo T, Grateau G, Leblond V, Godeau P, Piette JC (2005). AL cardiac amyloidosis and arterial thromboembolic events. Scand J Rheumatol.

